# Trematode Proteomics: Recent Advances and Future Directions

**DOI:** 10.3390/pathogens10030348

**Published:** 2021-03-16

**Authors:** Adam P. S. Bennett, Mark W. Robinson

**Affiliations:** School of Biological Sciences, Queen’s University Belfast, 19 Chlorine Gardens, Belfast BT9 5DL, Northern Ireland, UK; abennett12@qub.ac.uk

**Keywords:** trematode, host–parasite interactions, proteomics, secretome, mass spectrometry, excretory–secretory

## Abstract

Trematodes cause disease in millions of people worldwide, but the absence of commercial vaccines has led to an over-reliance on a handful of monotherapies to control infections. Since drug-resistant fluke populations are emerging, a deeper understanding of parasite biology and host interactions is required to identify new drug targets and immunogenic vaccine candidates. Mass spectrometry-based proteomics represents a key tool to that end. Recent studies have capitalised on the wider availability of annotated helminth genomes to achieve greater coverage of trematode proteomes and discover new aspects of the host–parasite relationship. This review focusses on these latest advances. These include how the protein components of fluke extracellular vesicles have given insight into their biogenesis and cellular interactions. In addition, how the integration of transcriptome/proteome datasets has revealed that the expression and secretion of selected families of liver fluke virulence factors and immunomodulators are regulated in accordance with parasite development and migration within the mammalian host. Furthermore, we discuss the use of immunoproteomics as a tool to identify vaccine candidates associated with protective antibody responses. Finally, we highlight how established and emerging technologies, such as laser microdissection and single-cell proteomics, could be exploited to resolve the protein profiles of discrete trematode tissues or cell types which, in combination with functional tools, could pinpoint optimal targets for fluke control.

## 1. Trematodiases: A Major Global Health Concern

Schistosomes and foodborne trematodes are thought to infect over 294 million people worldwide and result in the loss of 3.9 million disability-adjusted life years annually [1,2,3]. The diseases caused by these parasites (termed trematodiases) are endemic in 78 countries where they disproportionally affect people living in poverty and, accordingly, the World Health Organisation has named them amongst the world’s most neglected tropical diseases [4]. Additionally, three trematodes (*Clonorchis sinensis*, *Opisthorchis viverrini*, and *Schistosoma haematobium*) have been classified as group 1 carcinogens by the International Agency for Research on Cancer, and *S. mansoni*, *S. japonicum* and *O. felineus* have also been associated with inducing cancer (as reviewed by [5]).

Trematode life cycles involve a molluscan intermediate host that they use for asexual multiplication and a definitive host within which the flukes reach sexual maturity and produce eggs. Table 1 provides examples of trematodes of medical importance and their geographical distribution. Foodborne trematodes begin the intra-mammalian stages of their life cycle following the ingestion of undercooked fish, crustaceans, or semi-aquatic vegetation contaminated with infectious metacercariae (cysts). Upon excystment in the duodenum, the juvenile flukes migrate to the lung (e.g., *Paragonimus* spp.), liver/bile ducts (e.g., *C. sinensis*, *O. viverrini*, *Fasciola* spp.), proximal small intestine (e.g., *Fasciolopsis buski*), ileum (e.g., *Echinostoma* spp.), or colon (e.g., *Gastrodiscoides hominis*), where they mature and begin producing eggs that exit the host in the faeces (liver, lung, and intestinal flukes) or in the sputum (lung flukes only) [6]. Acute pathology can result from tissue damage caused by the migrating juveniles during the initial phases of infection, whilst chronic disease can be caused by the presence of the adult flukes inhabiting and interrupting the normal functioning of their target host organ [7]. In the case of schistosomes (blood flukes), such as *S. mansoni*, *S. haematobium*, *S. japonicum,* and *S. mekongi*, the infectious cercarial stage are found free-swimming in fresh water and directly penetrate the skin of individuals exposed to infested water during work, domestic, or recreational activities [6]. Prior to penetration, the cercariae lose their tails and form schistosomulae, which migrate via the bloodstream to the lungs and then the liver, where they develop into adult worms [8]. Unlike the foodborne trematodes, schistosomes are dioecious and have sexually dimorphic males and females that pair in the liver as a requisite for females to reach sexual maturity [9]. Then, the schistosome pairs migrate to the mesenteric venules of the intestine or, in the case of *S. haematobium*, the venous plexus of the bladder where they reside as adults [8]. The eggs of schistosomes cause the most acute pathology during infection. Female schistosomes deposit eggs on the blood vessel endothelium and the eggs’ secretions facilitate their passage into the lumen of the small intestine or bladder, depending on the species, allowing the eggs to exit the host in the faeces or urine [10]. However, worm pairs can also produce eggs, which become trapped in the tissue of host organs, such as the liver, where the eggs’ secretions induce the formation of granulomas, leading to fibrosis with pathological consequences [10,11].

Life cycles involving multiple hosts and developmental stages contribute to the challenge of treating trematodiases and controlling fluke infection rates in regions where they are endemic. Pharmaceutical interventions are reliant on just a few drugs, such as praziquantel and triclabendazole, but these do not prevent reinfection and require repeated usage. Triclabendazole, the only anthelmintic effective against immature *F. hepatica*, is also widely used to treat liver fluke infections in sheep and cattle, and its frequent application in the livestock industry has driven the selection of triclabendazole-resistant *F. hepatica* populations in several countries [12]. Whilst resistance to praziquantel, used to treat other trematode infections, is yet to emerge [12], praziquantel does not kill migrating schistosomulae, so a treated individual may still develop schistosomiasis and contribute to new infections in their community [13].

## 2. The Search for New Flukicidal Targets and Vaccine Candidates

Despite considerable research effort, no vaccine to prevent trematode infections has reached the levels of efficacy (approximately an 80% reduction in worm burden) required for commercialisation [14]. Vaccine development is complicated by the immunomodulatory mechanisms deployed by trematodes upon entering the mammalian host, which also limits natural protection to reinfection [14,15]. Therefore, new strategies to prevent and treat fluke infections are dependent on a deeper understanding of trematode–host interactions to identify the fluke-derived virulence factors and immunomodulators that are essential for fluke survival and, that, conceivably could be targeted by drugs or vaccines. To that end, studies employing mass spectrometry-based proteomics have been key to progressing our knowledge of the molecular cell biology of trematodes. Initial proteomics studies conducted from the early 2000s focussed on the characterisation of surface-exposed and secreted parasite proteins, often from adult flukes, due to the relative ease in isolating these molecules and their putative importance in the host–parasite interaction. This first generation of largely pre-genomic studies has provided a platform for the identification of vaccine candidates and has been extensively reviewed by various authors [16,17,18,19,20,21]. Building on this, recent studies have exploited the wider availability of annotated helminth genome/transcriptome resources to re-visit trematode proteomes from other tissues and developmental stages. This review will focus on these latest advances (summarised in Table 2), with an emphasis on novel approaches to study trematode proteomics, and highlight some future research perspectives.

**Table 1:** Examples of trematode species of medical importance (data obtained from references [7,22,23,24]).

	Source of Infection	Target Host Organ	Symptoms and Disease Associated with Infection	Geographical Distribution
**Foodborne trematodes**				
*Clonorchis sinensis*	Ingesting freshwater fish	Bile ducts	Acute: malaise, weakness, anorexia, flatulence, nausea, vomiting, abdominal pain, and diarrhoea Chronic: cholelithiasis, cholestasis, cholangitis, cholecystitis, biliary and liver abscesses, cirrhosis, pancreatitis, hepatitis, cholangiocarcinoma	China, Korea, Russia, Taiwan, Vietnam
*Fasciola hepatica*	Ingesting semi-aquatic vegetation, contaminated water or infected raw liver	Bile ducts	Acute: fever, abdominal pain, anorexia, flatulence, nausea, diarrhoea, urticaria, and coughing Chronic: cholelithiasis, jaundice, epigastric pain, nausea, fatty food intolerance, cholangitis, pancreatitis, cholecystitis	Worldwide (associated with the distribution of parasitised livestock)
*Opisthorchis viverrini*	Ingesting freshwater fish	Bile ducts	Acute: malaise, weakness, anorexia, flatulence, nausea, vomiting, abdominal pain, and diarrhoea Chronic: pyogenic cholangitis, biliary calculi, cholecystitis, cirrhosis of the liver, pancreatitis, and cholangiocarcinoma	Cambodia, Laos, Malaysia, Myanmar, Thailand, Vietnam
*Paragonimus westermani*	Ingesting crabs, crayfish or wild boar meat	Pulmonary tissue	Acute: cough, fever, bloody sputum, loss of appetite, chest pain, headache Chronic: consistent cough with brownish sputum, chest pain, night sweats	Cambodia, China, India, Japan, Korea, Laos, Malaysia, Nepal, Pakistan, Papua New Guinea, The Philippines, Southeast Siberia, Sri Lanka, Taiwan, Thailand, USA, Vietnam
*Fasciolopsis buski*	Ingesting semi-aquatic vegetation	Small intestine	Acute: diarrhoea, constipation, headache, flatulence, poor appetite, vomiting, abdominal pain, fever Chronic: oedema, anaemia, anorexia, vomiting, gastric pain, pallor, malnutrition, abdominal pain, nausea, bitemporal headache	Bangladesh, Cambodia, China, India, Indonesia, Japan, Korea, Laos, Malaysia, Myanmar, Nepal, Pakistan, Singapore, Taiwan, Thailand, The Philippines, Vietnam
**Blood flukes**				
*Schistosoma mansoni*	Contact with infested water	Mesenteric veins	Acute: myalgia, abdominal pain in the right upper quadrant, diarrhoea, fatigue, malaise, fever Chronic: intestinal disease, hepatosplenomegaly	Brazil, Burkina Faso, Burundi, Cameroon, Chad, Congo, Ivory Coast, Egypt, Ethiopia, Gambia, Ghana, Guinea, Kenya, Liberia, Madagascar, Malawi, Mali, Mauritania, Mozambique, Namibia, Nigeria, Oman, Puerto Rico, Rwanda, Saudi Arabia, Senegal, Sierra Leone, South Africa, Sudan, Suriname, Tanzania, Togo, Venezuela, Uganda, Yemen, Zambia, Zimbabwe
*Schistosoma japonicum*	Contact with infested water	Mesenteric veins	Acute: myalgia, abdominal pain in the right upper quadrant, diarrhoea, fatigue, malaise, fever Chronic: intestinal disease, hepatosplenomegaly	China, Indonesia, Taiwan, The Philippines
*Schistosoma haematobium*	Contact with infested water	Venous plexous of the bladder	Acute: myalgia, abdominal pain in the right upper quadrant, diarrhoea, fatigue, malaise, fever, haematuria Chronic: bladder pathology, cancer (squamous cell carcinoma, bladder cancer, vaginal cancer)	Angola, Burundi, Burkina Faso, Cameroon, Central Africa, Chad, Congo, Ivory Coast, Egypt, Ethiopia, Gabon, Ghana, Iran, Iraq, Kenya, Lebanon, Madagascar, Malagasy, Mauritius, Morocco, Mozambique, Namibia, Nigeria, Northern Syria, Réunion, Rwanda, Saudi Arabia, Senegal, Sudan, Tanzania, Togo, Uganda, Yemen, Zambia, Zimbabwe
*Schistosoma mekongi*	Contact with infested water	Mesenteric veins	Acute: myalgia, abdominal pain in the right upper quadrant, diarrhoea, fatigue, malaise, fever Chronic: intestinal disease, hepatosplenomegaly	Cambodia, Laos

## 3. A Closer Look at the Adult Fluke Secretome

Upon infection, trematodes must penetrate host tissue and overcome innate immune responses in order to reach their target organ system where they mature and produce eggs. This is achieved by the release of excretory/secretory products (ESP): a developmentally regulated cocktail of proteins and other molecules that act on host cells and tissue. ESP are readily released by parasites maintained in vitro; for instance, microgram amounts of protein can be collected from the culture medium of adult *F. hepatica* [16]. Therefore, adult fluke ESP has been targeted by a series of early proteomics studies, which led to the identification of a range of immunomodulators, virulence factors, and proteins involved in nutrient acquisition that facilitate parasite survival during chronic infection [16,19,21,25]. A recent characterisation of the secreted proteins of adult *C. sinensis* identified 175 proteins, including an abundance of metabolic enzymes and proteins that have roles in carcinogenic cellular pathways that may contribute to *C. sinensis*-induced cholangiocarcinoma in the liver [25]. Only one cathepsin-like cysteine peptidase was detected in the ESP of *C. sinensis* (cathepsin C; dipeptidyl peptidase I), and cathepsins have previously been shown to only constitute a minor proportion of the secretome of another Opisthorchiidae family member, *O. viverrini* [26]. This contrasts with *F. hepatica*, where cathepsin L peptidases account for >80% of the total protein secreted by adult fluke [27]. *Fasciola* spp. are obligate blood feeders and secrete cathepsins L into the gut lumen where they degrade haemoglobin into small peptides that can be absorbed by the gastrodermal cells before further intracellular degradation by cathepsin C and aminopeptidases (reviewed by [28]). Whilst blood may also feature in the diet of *C. sinensis* [29], the feeding habits of *O. viverrini* remain obscure [29], and these Opisthorchiidae may instead feed predominantly on exogenous free amino acids derived from host bile [30], which may account for the observed differences in the repertoire of digestive peptidases secreted by liver flukes.

Neuropathology and ocular manifestations have been reported in humans infected with *F. hepatica* [31], and proteomics analysis of the adult fluke secretome has suggested a mechanism of how neurological symptoms may be induced by flukes residing in the liver. In vitro, *F. hepatica* ESP was shown to bind human plasminogen, which is a zymogen found circulating in the bloodstream [32]. This binding enhanced the activation of plasminogen by tissue plasminogen activator to generate plasmin, which is a serine protease capable of degrading blood clots and the extracellular matrices of cells [32]. Mass spectrometry-based proteomics of plasminogen-binding proteins, recovered by ligand-blotting ESP separated on 2D electrophoresis gels with plasminogen, yielded 16 different proteins [32]. Amongst the most abundant plasminogen-binding proteins were isoforms of cathepsin L, enolase, and glutathione-S-transferase, indicating that these proteins may enhance the activation of plasmin. In vivo, this could impede blood clot formation to facilitate haematophagy, but the activation of plasmin in the bloodstream could impact sites distal to the liver, including the blood–brain barrier, where increased permeability could result in the neurological symptoms experienced during infection [32]. Such hypotheses could be tested by using in vitro models of the blood–brain barrier [33] to assess permeability following exposure to blood from patients infected by *F. hepatica*.

A new perspective in the secretion of trematode proteins followed the revelation that the ESP is compartmentalised into soluble and vesicular protein fractions [34]. Mass spectrometry profiling was used to show that over half of the proteins previously identified in the secretome of *E. caproni* and *F. hepatica* were associated with extracellular vesicles (EVs) that could be enriched by ultracentrifugation of the culture medium of parasites maintained in vitro [34]. The EV fraction of *F. hepatica*, *S. mansoni,* and *S. haematobium* adult secretions has been further separated into two populations with distinct protein compositions that pellet at either 15,000× *g* or 120,000× *g* by ultracentrifugation (named 15K or 120K EVs) [35,36,37]. Characterisation of the surface proteins of *F. hepatica* EVs was enabled by incubating the vesicles with a membrane-impermeable biotin reagent then performing liquid chromatography-tandem mass spectrometry (LC-MS/MS) on proteins isolated by streptavidin pulldown [38]. This revealed that proteases (including cathepsins), signalling proteins, and metabolic enzymes are displayed on the vesicle surface [38]. Cathepsins were similarly localised to the surface of *S. mansoni* EVs, leading to the suggestion that trematode EVs may participate in blood feeding [35,38]. Orthologues of EV biogenesis proteins including TSG101 and ALIX (also known as PDCD6IP) were also present on the surface of *F. hepatica* EVs [38]. Probing *F. hepatica* tissue sections with antibodies raised against these proteins showed that the sites of EV formation, trafficking, and release in the fluke included parenchymal cells, gastrodermal cells, the tegument, and the protonephridial system [39]. Moreover, incubating 15K and 120K EVs with antibodies against proteins displayed on the EV surface increased their internalisation by RAW264.7 macrophages compared to control EVs, suggesting that these epitopes are targets for antibody opsonisation [38]. However, the uptake of EV-enclosed molecules may be beneficial to the parasite. For example, the delivery of helminth defence molecule (an EV cargo protein) to the endolysosomal system would enable it to impair antigen processing by host macrophages or dendritic cells through the inhibition of lysosomal vATPase [38,40].

## 4. Characterisation of the Proteins Secreted by Early Life Stages

The proteins secreted by the early intra-mammalian life stages of liver and blood flukes account for much of the pathology during acute infection. Building on initial pre-genomic analysis of the secretome of *F. hepatica* newly excysted juveniles (NEJs), 21-day-old immature flukes, and adults [27], Cwiklinski et al. [41,42] re-visited these life stages using a combined transcriptomics and proteomics approach to capture the changes in the secretome as the parasite develops. In contrast to the micrograms of secreted protein that can be obtained from just 10 adult parasites [41], >3000 NEJs were required for collecting 10 µg of ESP for analysis by LC-MS/MS [41]. This enabled the identification of 135, 139, and 96 proteins secreted by NEJs at 1, 3, and 24 h post-excystment, respectively. Ten proteins constituted approximately 70% of the total protein for each time point [41], and these included cathepsins B (B1, B2, and B3) and L3 and a cysteine protease inhibitor (cystatin-1), which are involved in excystment and degradation of the cellular, interstitial, and muscular components of the intestinal lining [41,43]. Thioredoxin and peroxiredoxin were also amongst the most abundant secreted proteins and have both been implicated in inducing an M2 phenotype in macrophages [44,45], suggesting that the parasite begins to push the host toward a non-protective type 2 immune response within the first few hours of infection [41]. Four of the most abundant secretory proteins were uncharacterised, so their role in establishing infection remains to be determined. Aside from the most abundant molecules, the great diversity of proteins secreted by *F. hepatica* NEJs alludes to the mechanisms that enable them to overcome the various host immune defences and molecular barriers encountered when establishing infections in a greater number of terrestrial mammals than any other parasitic worm [41,46].

Cwiklinski et al. [42] subsequently focussed on the 21-day-old immature flukes that are responsible for the most tissue destruction as they migrate through the liver parenchyma. LC-MS/MS analysis detected 165 more secreted proteins than had been identified in the previous study [27,42], with a notable enrichment of cathepsin peptidases and cathepsin inhibitors (constituting 36% and 42% of total protein, respectively). This supports the view that immature flukes are dependent on cathepsins for the digestion of host tissue and blood during migration and feeding as the parasite’s gut begins to develop [27,42]. The three most abundant cathepsins belonged to cathepsin L2 and L3 clades, which display unique collagenolytic activity [43,47], whilst cathepsin L1 (specifically adapted to degrade host haemoglobin [48]) comprised a relatively minor proportion of the secretome compared to that of adult parasites [42]. The most abundant cathepsin inhibitor, a Kunitz-type 1 group member, represented 33% of total protein, highlighting the importance of the regulation of excessive damage to both host and parasite tissue before *F. hepatica* can become sexually mature in the bile ducts [42]. Of note, 92 of the proteins identified in the ESP of the immature fluke were shared with NEJs (350 proteins detected) and adults (227 proteins detected), and immunoproteomics analysis of these proteins could help to identify novel vaccine candidates effective against all of the parasite’s developmental stages.

Much of the morbidity associated with *Schistosoma* spp. infections stems from the immune response to proteins released by eggs trapped in host tissue. Recent comparative analysis by Carson et al. [49] using LC-MS/MS showed that the egg secretome differed markedly between species, with 266 proteins released by the eggs of *S. mansoni*, 90 from *S. japonicum* eggs, and 50 from *S. haematobium* eggs. Only 12 secreted egg proteins were common between the three species, and these included documented vaccine candidates (fatty acid-binding protein and fructose bi-phosphate aldolase) and antioxidant proteins that scavenge reactive oxygen species released by immune cells, thereby protecting the egg from damage within host granulomas [49]. Despite differences in individual proteins, gene ontology annotations revealed that the most highly represented functional groups had binding or catalytic activities or were part of metabolic processes. Of note, 40% of detected proteins across the three species were predicted to be exported in EVs, which may protect them from degradation by extracellular peptidases prior to delivery to host cells [49]. Although the secretion of EVs by trematode eggs has been reported [50,51], egg EVs are yet to be subjected to proteomics analysis. However, the application of new EV isolation techniques in trematode research [52,53] could facilitate the capture of sufficient material for mass spectrometry.

## 5. Immunoproteomics Identifies Secreted Proteins Specifically Recognised by the Host

Immunoproteomics involves the purification and mass-spectrometry based analysis of proteins that interact with the immune system. In particular, the capture of trematode ESP/tegument-exposed proteins by host antibodies has enabled the identification of antigenic vaccine candidates whilst filtering out exposed proteins that do not induce an immune response. Indonesian Thin Tail sheep mount an effective immune response to *Fasciola gigantica* infection, which is thought to be mediated through antibody-dependent cell-mediated cytotoxicity targeting exposed parasite proteins [54]. Cameron et al. [55] used an immunoproteomics approach to investigate the antigenic proteins driving this response (as well as their cross-reactivity in *Fasciola* spp.) by incubating adult *F. hepatica* with purified IgG obtained from Indonesian Thin Tail sheep pre-*F. gigantica* infection or at 4 weeks post-infection. The immunoprecipitate of antibodies bound to fluke proteins was then collected and analysed by LC-MS/MS. The 38 antigens that were significantly more abundant or unique in the post-infection immunoprecipitate represented the orthologues of *F. gigantica* proteins against which the sheep’s immune response is mounted [55]. Twenty of these antigens were predicted to be soluble secreted proteins, whilst 21 of the proteins were associated with *F. hepatica* 120K EVs [36,55], indicating the importance of EVs in host immune recognition and that impairing their function may impact fluke survival. Notably, cytosolic markers, indicative of non-specific shedding of parasite proteins, were not detected, and the recognised proteins did not correspond to those that were the most abundantly expressed, suggesting that the targeting of the protective immune response is specific to these antigens [55]. These observations may guide the appropriate selection of fluke antigens for future vaccine trials, which have historically used the most abundant antigens with varying levels of success [15].

Following these results, Huang et al. [56] similarly used an immunoproteomics approach to investigate the components of *F. gigantica* ESP that were recognised at later stages of infection in buffaloes. Antigenic targets were captured by incubation with buffalo serum obtained at 42, 70, and 98 days post-infection and then analysed by LC-MS/MS. The findings showed that the recognition of parasite proteins changed over the chronicity of infection, with thirteen, five, and seven proteins unique to the 42, 70, and 98 day time points, respectively [56]. This could suggest that the ESP components change as the parasites mature, as is the case in *F. hepatica* [27], or that the immune-privileged location of adult *F. gigantica* in the bile ducts and its release of immunoregulators influence the host’s ability to target specific proteins. Importantly, however, 17 proteins were consistently recognised by host sera, including cathepsins L, leucine aminopeptidase, and saposin-like protein 3 [56]. These proteins were also recognised by immune sera from Indonesian Thin Tail sheep at 28 days post-infection [55], indicating that the antibody response to these antigens is sustained as the infection progresses. Notably, the detected leucine aminopeptidase and cathepsin Ls have shown protective efficacy against *F. hepatica* in sheep vaccine trials (reviewed by McManus, 2020), and their immunogenicity in multiple host species and across both *F. hepatica* and *F. gigantica* at different developmental stages [55,56] supports their selection as vaccine candidates.

## 6. Insights from the Somatic Proteomes of Trematodes

Somatic proteomes of trematodes have been generated across different intra-mammalian life stages, providing insight into the biochemical processes that occur as the parasites develop. In addition to secreted egg proteins, the somatic egg proteins of *S. mekongi* have been shown to elicit an immune response and be targeted by sera from infected mice at 8 weeks post-infection [57]. Immunoproteomics demonstrated that 21 somatic egg proteins were recognised by mouse antibodies, including cytoskeletal proteins (actin), chaperones (heat shock protein 70), and lipolytic enzymes (ceramidases) [57]. Interestingly, when the somatic egg extracts were probed with sera from infected humans, only two of the proteins recognised (out of 16) were the same as those bound by mouse sera (DNA replication factor Cdt1 and heat shock protein 70), highlighting the need for caution when translating information obtained from time-controlled murine models of schistosome infection to human patients [57].

Somatic proteomes of adult trematodes have been used to investigate how polyparasitism can affect the expression of fluke proteins. Pairing of male and female adult schistosomes is required for the differentiation of the female reproductive organs, and unpaired schistosome females do not reach complete reproductive maturity or produce normal eggs [9]. To understand the proteomic basis underlying these observations, somatic lysates of 25-day-old *S. japonicum* were recovered from either single-sex or bisexually infected mice and proteins were quantified with isobaric tags for relative and absolute quantitation (iTRAQ)-coupled LC-MS/MS [58]. A total of 1477 distinct proteins were differentially expressed between paired and unpaired females, providing a list of candidate proteins required for female maturation. Bioinformatics analyses showed an enrichment of proteins belonging to pathways involved in eggshell production, protein synthesis, and glucose metabolism in paired female schistosomes, which may explain their enhanced growth and egg production compared to unpaired worms [58]. Conversely, proteins involved in locomotion, such as myosin and tropomyosin, were more highly expressed in unpaired females, which move freely in the bloodstream whilst continuing their search for a mate, whereas paired females decrease their movement in the male gynaecophoral canal [58].

Mass spectrometry analysis of fluke somatic proteomes could also be used in clinical diagnoses, or epidemiological surveys, to distinguish closely related species. *F. hepatica* and *F. gigantica* can be difficult to distinguish based on morphology alone, and their identification often requires laborious typing based on PCR and the sequencing of various nuclear and mitochondrial gene markers (e.g., [59]). Matrix-assisted laser desorption/ionisation time-of-flight (MALDI-TOF) mass spectrometry is already used in clinical settings for microbial diagnoses [60,61] and could be used to expand the molecular tools available for parasite identification. Sy et al. [62] subjected the posterior region of *F. hepatica* or *F. gigantica* to mass spectrometry-based analysis to construct species-specific spectral profiles, which were then included in a helminth identification database. To validate the database, 78 unidentified samples from different geographical locations were analysed by MALDI-TOF mass spectrometry. This resulted in the correct identification of 98.7% of *F. gigantica* and 100% of *F. hepatica* samples compared to established molecular methods. Although the unidentified sample numbers were small (e.g., only four *F. hepatica* samples were tested), this study provided initial proof-of-concept for the use of MALDI-TOF mass spectrometry as a screening tool to differentiate between helminth species [62]. Whilst useful from an epidemiological standpoint to assess the distribution of each species in geographic regions, diagnosing *Fasciola* spp. would not affect clinical treatment at present [63]. However, fine-tuning this approach to recognise spectra unique to drug-resistant flukes or hybrid species could inform future treatment choices and make this a clinically relevant tool, especially if it could be adapted to trematode eggs recovered from patients’ stool samples without the need for invasive clinical procedures. However, it should be noted that trematode eggs do not always appear in faeces during human infection [64].

## 7. Subproteome Level Analysis of Trematode Tissues

Somatic (i.e., whole fluke) proteomes are of limited use when investigating protein-driven processes intrinsic to individual tissues. To circumvent this, a series of targeted proteomics studies in trematodes have so far focussed on the tegument, a major host–parasite interface, which can be detached by subjecting the worms to freeze–thaw cycles or by treating them with detergent to yield an extract enriched in tegumental proteins. Additionally, studies have recovered surface exposed tegumental proteins through trypsin shaving or phospholipase C treatment to directly release proteins for analysis, or via the biotinylation of live flukes followed by streptavidin pulldown. These techniques have become established in trematode proteomics research and have defined the tegument as a dynamic and protective barrier to host attack, as has been reviewed previously [16,18,20].

A new approach to tegument proteomics has been applied by Swan et al. [65], who investigated which *F. hepatica* glycoproteins were the binding partners of host galectins. The outer layer of the tegumental syncytium is highly glycosylated [66], and parasite glycans are potential targets of host galectins which function in both the innate and adaptive immune response [67]. Two ruminant-specific galectins, LGALS-11 and LGALS-14, are upregulated following *F. hepatica* infection and are present in the bile of infected, but not uninfected, sheep [68]. To better understand their role in the host–parasite interaction, the two galectins were immobilised on sepharose beads; then, glycoprotein capture was performed with a *F. hepatica* tegument extract or a protein extract generated from the fluke body with the tegument removed [65]. Following elution, LC-MS/MS was performed on bound parasite proteins to identify the galectins’ ligands. The most abundant protein bound by both galectins in all the extracts was an uncharacterised protein that contained a C-type lectin domain [65]. This protein has also been detected on the tegumental surface [69] and is associated with *F. hepatica* EVs [36] and therefore represents an immunogenic protein exposed to the host that warrants further investigation into its function [65]. Another set of proteins that are relatively uncharacterised in fluke infections, but were highly abundant in the bound fractions, are the natterin family of proteins that contain a DM9 domain [65]. In fish venom, these proteins are thought to induce vasodilation and oedema [70], which could also be beneficial to a blood-feeding parasite; however, the bound *F. hepatica* natterins lacked predicted glycosylation sites and may instead have been bound due to their interactions with other parasite glycoproteins, which is a limitation of identifying the targets of host lectins using this approach [65].

**Table 2:** A summary of the recent trematode proteomics studies included in this review.

Trematode and Life Stage	Study	Proteomics Techniques	Reference
Adult *Clonorchis sinensis*	Characterisation the ESP, tegument, and tegumental surface proteins	Freeze–thaw enrichment of tegument proteins, biotinylation of tegument surface proteins, LC-MS/MS	[25]
Adult *Fasciola hepatica*	Identification of plasminogen-binding proteins in the ESP	2DE, ligand-blotting ESP with plasminogen, MALDI-TOF/TOF MS, LC-MS/MS	[32]
Adult *Fasciola hepatica*	Characterisation of extracellular vesicle surface proteins	Biotinylation of extracellular vesicle surface proteins, LC-MS/MS	[38]
*Fasciola hepatica* NEJs	Comparison of the ESP of NEJs at 1, 3, and 24 h post-excystment	SDS-PAGE, LC-MS/MS	[41]
*Fasciola hepatica* immature flukes	Characterisation of the somatic proteome and ESP and comparison with the ESP of NEJs and adults.	LC-MS/MS	[42]
The eggs of *Schistosoma mansoni*, *Schistosoma japonicum* and *Schistosoma haematobium*	Comparison of the ESP released by the eggs of the three species	LC-MS/MS	[49]
Adult *Fasciola hepatica*	Identification of antigenic fluke proteins recognised by purified IgG obtained from Indonesian Thin Tail sheep at 4 weeks post-*Fasciola gigantica* infection	Immunoprecipitation, LC-MS/MS	[55]
Adult *Fasciola gigantica*	Identification of antigenic ESP recognised by buffalo sera at 42, 70, and 98 days post-infection	Immunoprecipitation, LC-MS/MS	[56]
*Schistosoma mekongi* eggs	Identification of somatic egg proteins recognised by infected mouse and human sera	2DE-immunoblotting, LC-MS/MS	[57]
Adult female *Schistosoma japonicum*	Comparison of somatic proteomes of flukes from single-sex and bisexual infections	iTRAQ-coupled LC-MS/MS	[58]
*Fasciola hepatica* and *Fasciola gigantica* adults	MS-based identification of fluke species based on species-specific spectral profiles	MALDI-TOF MS	[62]
Adult *Fasciola hepatica*	Identifying the binding partners of host galectins upregulated during infection	Detergent-based enrichment of tegumental proteins, glycoprotein capture with immobilised galectins, LC-MS/MS	[65]
Adult male *Schistosoma mansoni*	Characterisation of oesophageal gland proteins	LC-MS/MS, QconCAT	[71]

Abbreviations: 2DE, two-dimensional gel electrophoresis; ESP, excretory/secretory products; iTRAQ, isobaric tags for relative and absolute quantitation; liquid chromatography-tandem mass spectrometry; MALDI, matrix-assisted laser desorption/ionisation; MS, mass spectrometry; SDS-PAGE, sodium dodecyl sulfate-polyacrylamide gel electrophoresis; TOF, time-of-flight; NEJ, newly excysted juvenile.

The composition of trematode internal tissue proteomes remains relatively undefined, and their isolation requires the application new techniques. The oesophagus and gut of schistosomes are targeted by a protective humoral immune response in rhesus macaques that kills the parasites by impairing their ability to lyse and digest blood cells, thus causing starvation [72]. Since the relative abundance of oesophageal gland proteins is too low to be detected in preparations of somatic worm extracts [73], microdissection was used to enrich oesophageal gland tissue by removing the neck region of adult male *S. mansoni* from the rest of the body [71]. The posterior end of the fluke was also excised for comparative analysis, and both tissues were subjected to LC-MS/MS and label free quantification [71]. Additionally, nine proteins shown to be expressed in the oesophageal glands by in situ hybridisation were targeted for absolute quantification using QconCAT technology, which quantifies proteins relative to internal reference peptide standards [74]. This approach revealed that the nine target proteins were found to constitute only 0.5% of the total protein in the extract. However, when presented as protein copies per oesophageal gland cell (to account for the presence of other cell types in the extract), four of these proteins (MEG-12, VAL-7, MEG-4.2, and MEG-4.1) were highly abundant, with between 4.5 and 245 million copies per cell, indicating that they are key components at this interface with the blood [71]. This approach also enabled the detection of multiple MEG isoforms, with alternative splicing thought to be employed to enable antigenic variation and immune evasion [71]. Furthermore, comparing the protein abundance in the oesophageal gland extract relative to the posterior body extract showed that the glands were specifically enriched in proteins involved in glycerophospholipid and sphingolipid metabolism, whilst the posterior end showed an over-representation of cysteine peptidases. There was also a significant enrichment of proteins involved in vesicle trafficking and secretion in the gland extract, highlighting the importance of protein secretion in this tissue to begin the blood processing cascade [71].

## 8. Future Directions in Trematode Proteomics Research

**Figure 1:**
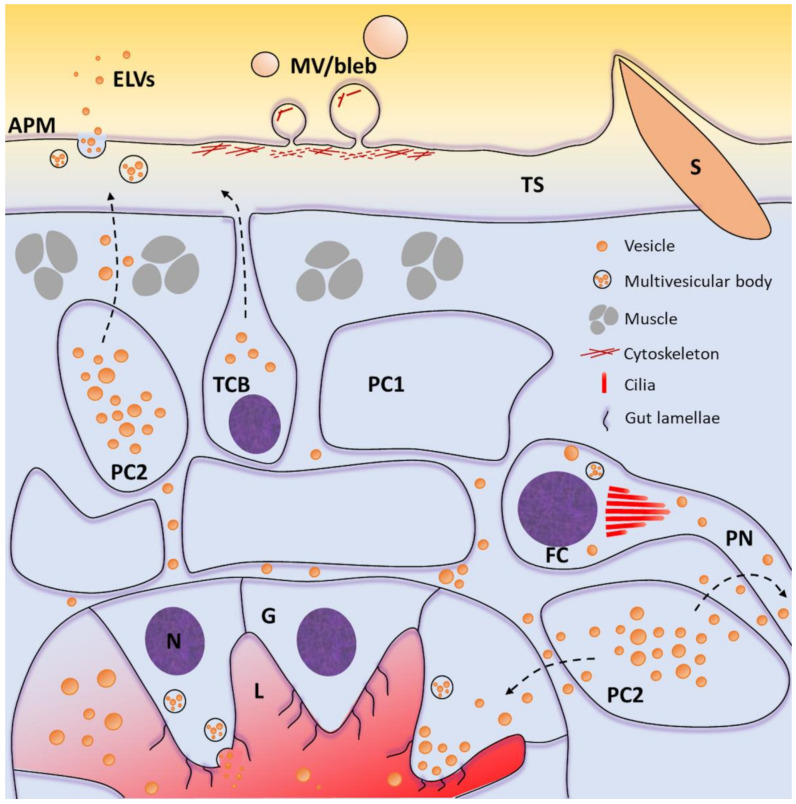
Schematic representation of the major host interfaces of trematodes and highlighting key cells/tissues for future proteomics analysis. The tegumental syncytium (TS) and gastrodermis (G) represent tissues amenable to isolation by laser microdissection for subproteome level analysis. Whilst technically challenging, heterogenous cells from internal fluke tissues, including tegumental cell bodies (TCB), type 1 (PC1) and type 2 (PC2) parenchymal cells, and flame cells (FC) could be dissociated into single-cell suspensions and sorted into individual cell types for single-cell proteomics. Diverse populations of extracellular vesicles (including microvesicles/blebs and exosome-like vesicles) released from distinct cell types could be separated using new technologies to define the protein profiles and function of each extracellular vesicle subtype. APM, apical plasma membrane; ELVs, exosome-like vesicles; L, gut lumen; MV, microvesicle; N, nucleus; PN, protonephridial duct; S, spine. Dashed arrows; potential routes of vesicle trafficking.

The major trematode interfaces with the host, and key cell types for future targeted proteomics analysis, are summarised in Figure 1. To define the proteome of individual trematode tissues, more precise isolation techniques are required than those used to date, which only enrich for proteins expressed in each tissue. Moreover, the current detergent and freeze–thaw tegument detachment methods are prone to contamination from internal cellular components and secreted proteins, leading to ambiguity around the source of some proteins in mass spectrometry datasets [69]. Laser microdissection of fluke tissue from histological sections offers a more precise isolation technique and has already been employed to recover the gastrodermis and reproductive organs of schistosomes for transcriptional profiling [75,76]. The sensitivity of modern mass spectrometers also makes tissue recovered by laser microdissection amenable to proteomics analysis, and this approach has been successfully used to characterise the gut, tegument, and parenchymal tissue of the monogenean *Eudiplozoon nipponicum* [77].

Future research may also move away from bulk proteomics experiments (which yield the average protein composition of a range of cells) to single-cell proteomics, which would reveal the cellular heterogeneity that exists in parasite tissues. Single-cell transcriptomics has already been employed in schistosomes to characterise different cell types based on their gene expression patterns and to track cell fates [78,79,80]. Single-cell proteomics by mass spectrometry (SCP-MS) is based on a similar starting principle where tissue is dissociated into a single-cell suspension from which cells are separated using fluorescent-activated cell sorting (FACS) based on cell size and shape or specific surface proteins [81]. Then, proteins are extracted, digested with trypsin, labelled with isobaric tags (iTRAQ), and analysed by LC-MS/MS [81]. This approach could be used to elucidate protein abundance and post-translational status within specialised fluke cells with important roles in nutrient acquisition, host defence, or reproduction (e.g., gastrodermal cells, tegumental cells, or vitelline cells).

Further resolution of protein expression within the cell could be gained by hyperplexed localisation of organelle proteins by isotype tagging (hyperLOPIT) of the trematode single cell suspensions. This technique has recently been applied to *Toxoplasma gondii* and involves the separation of organelles by differential centrifugation and then analysis of all the cellular compartments simultaneously in a single mass spectrometry experiment, thus enabling spatial subcellular proteomics analysis [82]. Such an approach could complement functional studies (e.g., RNA interference) in helping to identify phenotypes for uncharacterised parasite proteins, which remain a “black box” in trematode proteome datasets due to their lack of sequence similarity to known proteins [83]. Similarly, the further fractionation of trematode EV populations into more defined subpopulations using new technologies such as asymmetric flow field-flow fractionation [84] could help elucidate the effects of discrete EV subgroups on different host cell types and narrow down the number of candidate EV proteins that could be blocked with antibodies or drugs for therapeutic purposes.

A comparative study of helminth EV proteome datasets has already indicated that shared EV biogenesis mechanisms may operate in trematodes [85], and resolving the protein composition of fluke EV subsets could lead to the identification of common, essential biogenesis pathway members that, if inhibited, prevent EV release, a precedent for which has already been demonstrated in *F. hepatica* [39]. Absolute quantification using QconCAT technology coupled with LC-MS/MS could also be used to determine the levels of targeted EV proteins in host fluids (which would be present at relatively low levels compared to host proteins) as a means to track the release of parasite proteins during infection and under different conditions, such as following drug treatment. The trematode research community has been very successful in identifying proteins expressed by a number of important species via mass spectrometry-based proteomics techniques. The challenge now is to transition from simply “cataloguing” these proteins to generating a “moving picture” of the trematode proteome that not only charts protein expression at temporal (throughout the parasite’s life-cycle) and spatial (cellular and subcellular) levels but also addresses protein function. Key to this will be the continued use of emerging mass spectrometry-based tools in conjunction with bioimaging, RNAi-induced gene silencing [86], and CRISPR/Cas9 genome editing techniques [87,88] to investigate the roles of trematode proteins during infection. This combined approach will provide further insight into trematode–host interactions and bring the next generation of vaccine candidates and flukicidal drug targets into sharp focus.

## Data Availability

Data sharing not applicable.
